# Anidulafungin Treatment Blocks the Sexual Cycle of Pneumocystis murina and Prevents Growth and Survival without Rescue by an Alternative Mode of Replication

**DOI:** 10.1128/spectrum.02906-22

**Published:** 2022-10-26

**Authors:** Melanie T. Cushion, Alan Ashbaugh, Steven G. Sayson, Christopher Mosley, Philippe M. Hauser

**Affiliations:** a Medical Research Service, Cincinnati Veterans Affairs Medical Center, Cincinnati, Ohio, USA; b Department of Internal Medicine, University of Cincinnati College of Medicine, Cincinnati, Ohio, USA; c Institute of Microbiology, Lausanne University Hospital and University of Lausanne, Lausanne, Switzerland; University of Minnesota Medical School

**Keywords:** echinocandin, *Pneumocystis*, *Pneumocystis* pneumonia, fungal sexual reproduction, fungal asexual reproduction, asci, echinocandins

## Abstract

The proposed life cycle of fungi in the genus Pneumocystis has typically included both an asexual cycle via binary fission and a sexual cycle. Until recently, the strategy used for sexual replication was largely unknown, but genomic and functional assays now support a mode known as primary homothallism (self-fertilization). The question of whether an asexual cycle contributes to the growth of these fungi remains. Treatment of Pneumocystis pneumonia in immunosuppressed rodent models with the class of drugs known as echinocandins is challenging the historical concept of asexual replication. The echinocandins target 1,3-β-D-glucan (BG) synthesis resulting in death for most fungi. Because Pneumocystis species have both non-BG expressing life cycle stages (trophic forms) and BG-expressing asci, treatment with anidulafungin and caspofungin resulted in elimination of asci, with large numbers of non-BG expressing organisms remaining in the lungs. Transcriptional analyses of anidulafungin treated Pneumocystis murina-infected lungs indicated that these agents were blocking the sexual cycle. In the present study, we explored whether there was an asexual or alternative method of replication that could rescue P. murina survival and growth in the context of anidulafungin treatment. The effects of anidulafungin treatment on early events in the sexual cycle were investigated by RT-qPCR targeting specific mating genes, including *mam2*, *map3, matMi, matPi,* and *matMc*. Results from the *in vivo* and gene expression studies clearly indicated there was no rescue by an asexual cycle, supporting these fungi’s reliance on the sexual cycle for survival and growth. Dysregulation of mating-associated genes showed that anidulafungin induced effects early in the mating process.

**IMPORTANCE** The concept of a sexually obligate fungus is unique among human fungal pathogens. This reliance can be exploited for drug development and here we show a proof of principle for this unusual target. Most human fungal pathogens eschew the mammalian environment with its battery of immune responses. Pneumocystis appear to have evolved to survive in such an environment, perhaps by using sexual replication to help in DNA repair and to introduce genetic variation in its major surface antigen family because the lung is the primary environment of these pathogens. The concept of primary homothallism fits well into its chosen ecosystem, with ready mating partners expressing both mating type receptors, and a sexual cycle that can introduce beneficial genetic variation without the need for outbreeding.

## INTRODUCTION

Echinocandins are a relatively new class of drugs that were designed to treat fungal infections. The target of these compounds is 1,3-β-D-glucan (BG) synthesis and this linear glucose polymer is found in most, but not all fungal cell walls. Previously, our laboratory reported the effects of anidulafungin, caspofungin, and micafungin against Pneumocystis pneumonia in prophylactic and treatment animal models of the infection (mice and rats) (1). We observed that asci were depleted to microscopically undetectable levels after treatment with the echinocandins, but large numbers of organisms that were not asci, remained in the lungs. This was in keeping with the fact that Pneumocystis species (spp.) are bi-phasic in expression of BG, with only the asci expressing BG while other life cycle stages do not. Taking advantage of the absence of asci, we showed that these echinocandin-treated mice were incapable of transmitting the infection via the airborne route, providing strong evidence that asci are the agents of transmission. Furthermore, the Pneumocystis murina in the lungs of the treated mice were inoculated into untreated immunosuppressed mice resulting in infections replete with asci, showing that these non-BG expressing organisms were viable, even after 3 weeks of therapy. The ability to prevent the infection was also explored with anidulafungin and caspofungin treatment at low doses. Both were able to prevent severe disease, although small numbers of non-BG expressing forms were present, without detectable asci.

Although the complete life cycle of Pneumocystis species spp. is not yet known, several new concepts have emerged in the past few years that have enhanced our understanding of Pneumocystis spp. growth, survival, and transmission. In the mammalian lung, unicellular forms, called “trophic forms,” possess three MAT genes that are sufficient to trigger mating and meiosis through primary homothallism, the mode that involves a single, self-compatible mating type that can enter the sexual phase on its own (2–4). While there are different mechanisms of primary homothallism, genomic and functional studies have shown that the two mating type receptors, Mam2p and Map3p, are expressed on a single trophic form, facilitating mating with any other trophic form (2, 3). This mode of sexual reproduction could be advantageous for Pneumocystis spp. which are relegated to the lung as their primary ecosystem and only appear to be exposed to the external environment when asci leave one host to infect another.

The hypothetical life cycle of Pneumocystis spp. has almost always included an asexual mode via binary fission, along with the sexual cycle (5). Recently, however, the existence of an asexual cycle for Pneumocystis species spp. has been called into question (4, 6, 7). The purpose of the present studies was to explore whether there was an alternative mode of replication used by P. murina to establish infection when the sexual cycle was blocked by anidulafungin. In the first study, the prophylactic model of infection was used. In the second study, we asked whether the organisms remaining after 3 weeks of anidulafungin treatment could survive and replicate when transferred to immunosuppressed mice receiving anidulafungin. Though results from the prophylactic study raised the possibility of an alternative mode of replication, the inoculation of anidulafungin-treated P. murina did not survive in anidulafungin treated recipients. Thus, we conclude that in these experimental models of infection, an alternative or asexual cycle could not replace the sexual cycle for growth and survival.

## RESULTS

### Prophylaxis with anidulafungin dramatically inhibited P. murina pneumonia.

**FIG 1:**
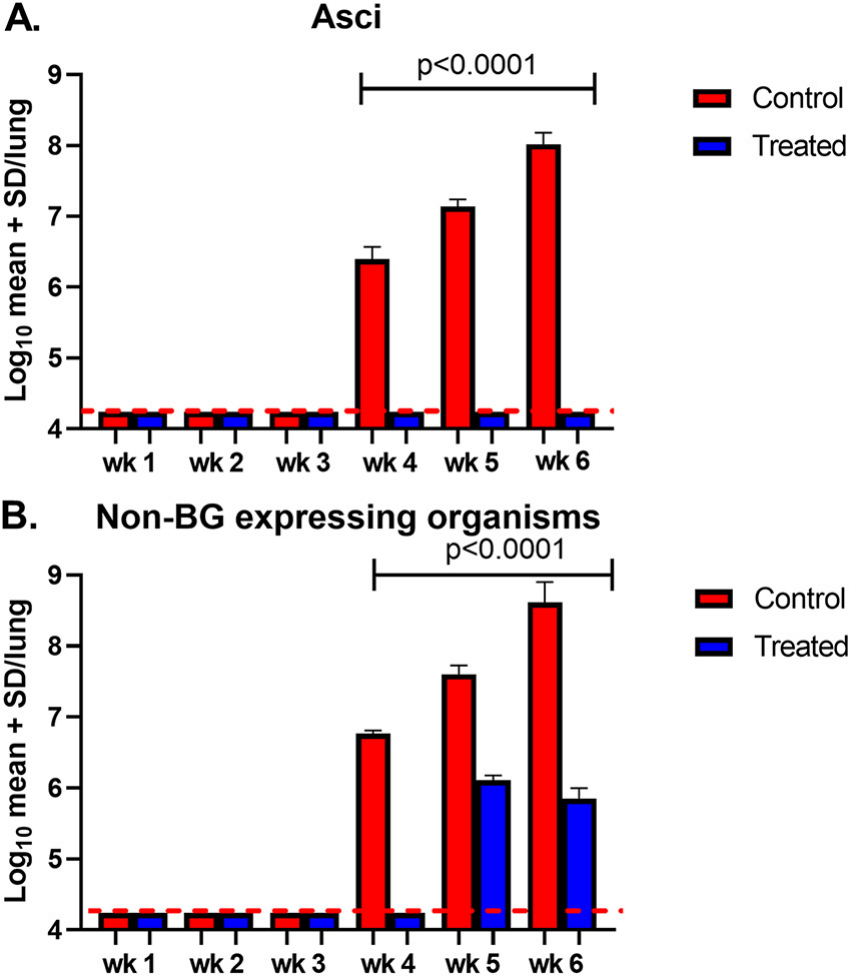
Asci and non-BG expressing burdens in mice untreated or treated with 1 mg/kg/wk anidulafungin. (A) Asci burdens. (B) Non-BG expressing organisms (nuclei). Red bars = controls; blue bars = treated. Mice were sacrificed weekly for 6 weeks. At each time point, three mouse lungs were homogenized for quantification of P. murina asci and nuclei (non-BG expressing organisms). Capped lines indicate significant differences between control and treated groups. Red dotted line indicates limit of microscopic detection (log_10_ 4.24).

We asked whether inhibition of asci production by anidulafungin treatment could prevent establishment of P. murina pneumonia in immunosuppressed mice. In previous studies, we showed that anidulafungin treatment could suppress the pneumonia when given prophylactically, although small numbers of non-BG producing organisms remained (1). In the present experiment, untreated immunosuppressed mice developed a moderate infection after 4 weeks and severe pneumonia after 5 to 6 weeks of immunosuppression postinoculation of P. murina (Fig. 1A and B, red bars), as expected for both asci and non-BG expressing organisms. In contrast, those mice that were treated with anidulafungin lagged in numbers of non-BG expressing organisms (Fig. 1B, blue bars) and did not develop microscopically detectable asci (Fig. 1A). Previous studies that used the prophylactic mouse model revealed microscopically detectable asci and non-BG forms at the terminus of the study (6 weeks) when administered anidulafungin 1 mg/kg once a week for the 6 weeks (1). In this study, we observed an increase in numbers of non-BG organisms at weeks 5 to 6 in those mice that were treated with anidulafungin (Fig. 1B), although they were statistically much less than the numbers in untreated control mice. Asci were undetectable by microscopic methods in the treated mice. This finding may suggest that replication of some kind was occurring. To determine whether there may be cryptic asci production, the same lung homogenates were evaluated for BG content which is only found in asci.

**FIG 2:**
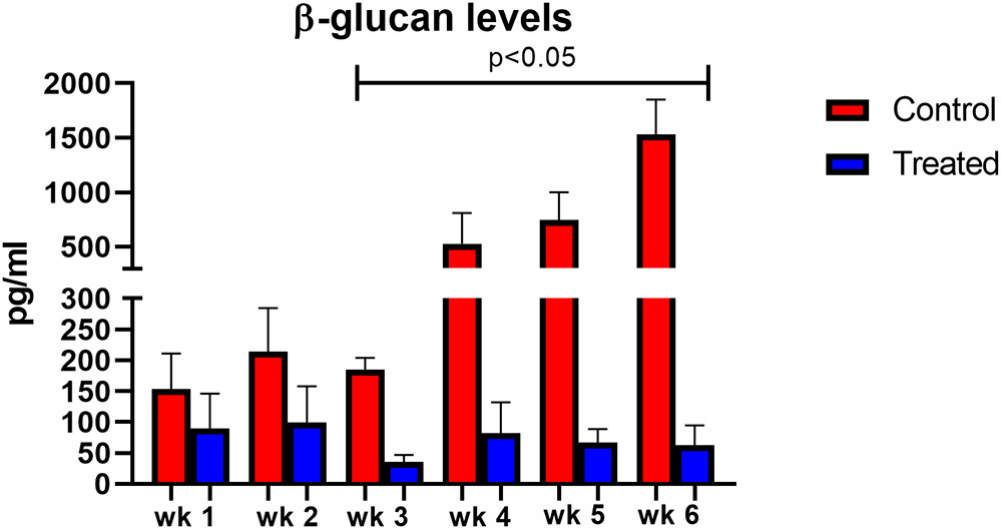
BG levels from anidulafungin-treated (1 mg/kg/wk) and untreated mice. Red bars = controls; blue bars = treated. Mice were sacrificed weekly for 6 weeks. At each time point, three mouse lungs were homogenized for quantification of BG. Capped lines indicate significant differences between treated and untreated control mice.

During the 6 weeks of immunosuppression, BG was measured each week using the GlucaTell assay kit (Fig. 2). In untreated mice, (red bars) the BG content increased over time with significant increases after 3 weeks versus treated mice (blue bars) (150 to 1,500 pg/mL in untreated mice versus 75 to 25 pg/mL in treated mice). The lack of concordance between the increase in numbers of non-BG organisms at 5 and 6 weeks by microscopic enumeration (Fig. 1B) and the static expression of BG (Fig. 2) could suggest a small amount of asci production during the infection, reflects a small number of P. murina asci that were resistant to the echinocandin, represent dead asci that have not yet been cleared, or residual BG in the lungs without asci.

### Gene expression in the echinocandin-treated and -untreated mice in the prophylaxis model.

**FIG 3:**
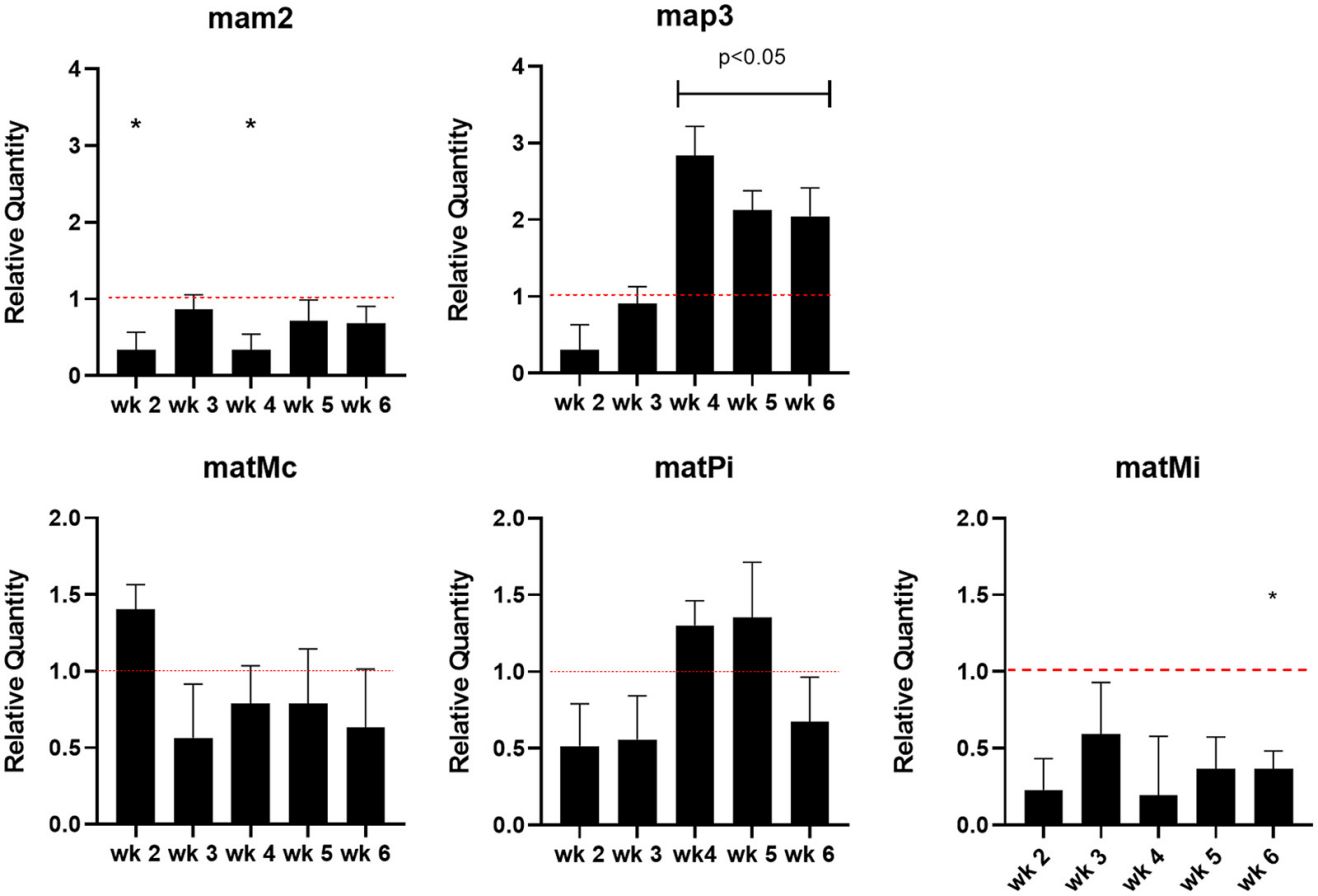
Differential regulation of mating-related genes of P. murina. Gene expression of sex-related genes in the lungs of anidulafungin-treated mice from 2 to 6 weeks after inoculation. *Mam2* and *map3* genes encode the receptors for the plus and minus pheromones of P. murina. *MatMc* encodes the transcription factor that initiates sexual differentiation into the minus (M) mating type. The *matPi* gene also encodes a transcription factor with the cofactor *matMi.* RT-qPCR was conducted as described in the Materials and Methods. The threshold cycle (Δ*CT*) value between the validation gene and the thymidylate synthase (TS) gene was calculated by subtracting the average *CT* value of the single-copy reference gene from the average *CT* value of the validation gene. ΔΔ*CT* values were calculated by subtracting the Δ*CT* value of the reference sample from the Δ*CT* value of the experimental sample. Relative quantity is shown as 2^_ΔΔ^*^CT^*. Red dotted line indicates baseline expression in untreated control mice. Significance was assessed using multiple *t* test analysis by GraphPad Prism v.8. Capped lines and asterisks indicate significant differences between treated and untreated control mice.

**FIG 4:**
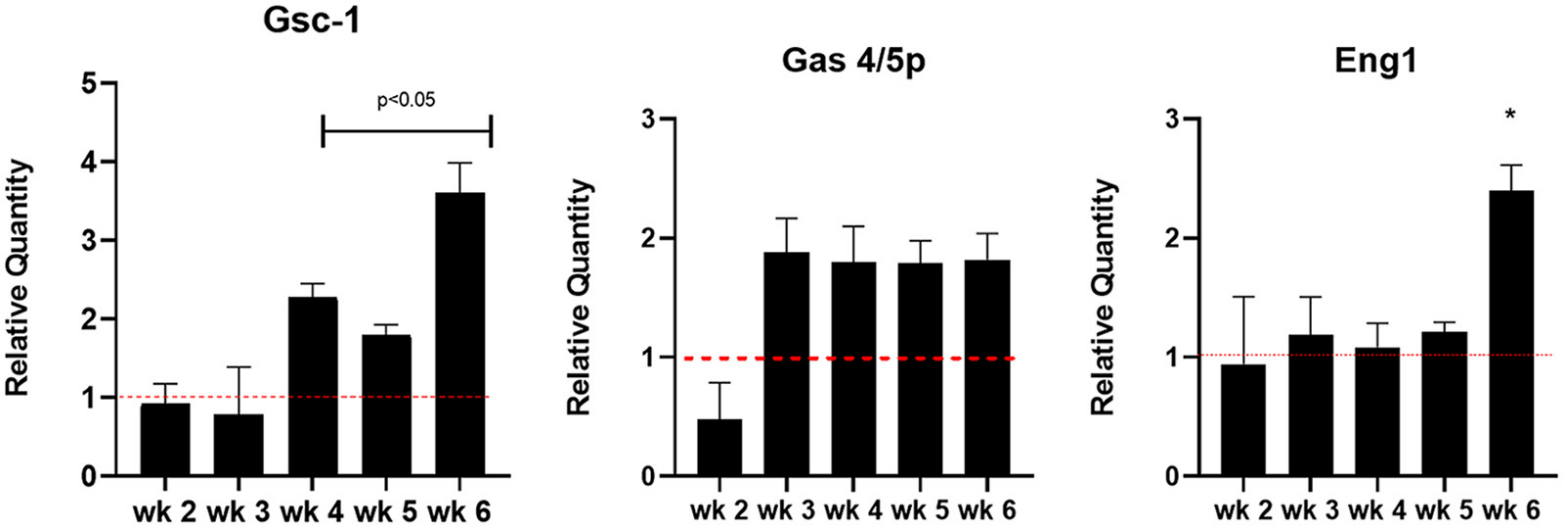
Differential gene regulation of genes associated with BG synthesis and remodeling. RT-qPCR was conducted as described in the Materials and Methods. The threshold cycle (Δ*CT*) value between the validation gene and the thymidylate synthase (TS) gene was calculated by subtracting the average *CT* value of the single-copy reference gene from the average *CT* value of the validation gene. ΔΔ*CT* values were calculated by subtracting the Δ*CT* value of the reference sample from the Δ*CT* value of the experimental sample. Relative quantity is shown as 2^_ΔΔ^*^CT^*. Red dotted line indicates baseline expression in untreated control mice. Significance was assessed using multiple *t* test analysis by GraphPad Prism v.8. Capped lines and asterisks indicate significant differences between treated and untreated control mice.

**FIG 5:**
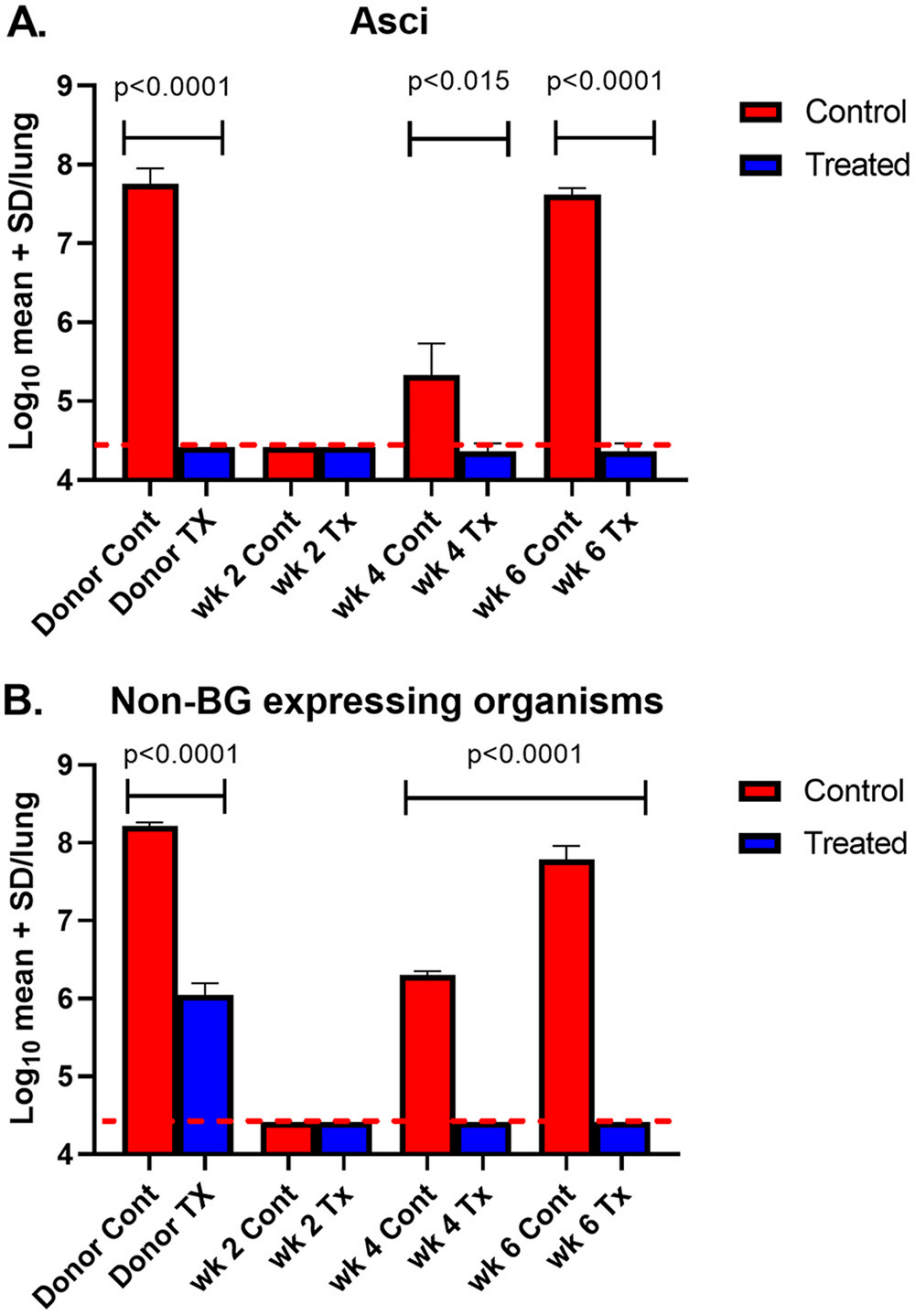
Inoculation of untreated and anidulafungin-treated P. murina into untreated and treated recipient mice. Asci and non-BG expressing organism burdens from untreated-immunosuppressed mice that received untreated P. murina (red bars) or from mice that were anidulafungin treated that received P. murina from mice treated with anidulafungin (blue bars). (A) Asci burdens from untreated donor mice “Donor Cont” (red bar) and from treated donor mice “Donor TX” (blue bar), then at 2-, 4-, and 6-weeks postinoculation. (B) Non-BG expressing organism burdens from donor control mice (red bar) and treated donor mice (blue bar), then at 2-, 4-, and 6-weeks postinoculation. Capped lines indicate significant differences between control and treated groups. Red dotted line indicates limit of microscopic detection (log_10_ 4.24).

Next, we sought to evaluate the relative abundance of *matMc*, *matMi*, *matPi*, *mam2*, and *mam3* gene expression in the anidulafungin-treated mice. (Fig. 3). These genes are expressed during infection and are associated with primary homothallism, the mode of sexual reproduction in Pneumocystis spp. Formation of asci was evaluated by expression of *gsc-1* gene, which mediates BG synthesis (Fig. 4). *Gas4/5* and *eng1* expression levels were evaluated, as they are involved in BG regulation (Fig. 5).

The expression of one of the two mating type receptors, *mam2*, was significantly reduced throughout the 6 weeks of treatment Fig. 3 (the dotted line indicates no differential expression), while the other mating type receptor gene, *map* 3 was significantly increased at 3 weeks of infection and thereafter. Of note, we identified the same downregulation of the *mam2* gene after anidulafungin treatment in our previous publication (8). This dysregulation of the two mating type receptors could indicate a mechanism whereby sexual reproduction is inhibited if both receptors are needed in a model of primary homothallism. It is also interesting to note that although the *mam2* gene was downregulated compared with the reference gene, it did show statistically significant differences at weeks 2 and 4 versus week 3 where it had increased expression relative to the other weeks. At week 4, the *map3* gene expression was at its peak and it is tempting to speculate that this dysregulation may contribute to the lack of successful sexual reproduction.

Based on the mating process in the closely related S. pombe, *matMc* gene encodes a transcription factor which facilitates the conversion to the minus (M) mating type whereas the *matPi* gene works with the *matM*i gene to activate the *mei3* gene which is the single gene whose expression is sufficient and required to trigger meiosis (4). However, the mechanism by which Pneumocystis spp. undergoes these processes remains unclear, because orthologs to *mei3* nor to the gene responsible for differentiation into the plus (P) mating type, *matPc*, were identified in the genome (4). In Pneumocystis spp., *matMi* and *matMc* are located on the same DNA strand in the genome and share a promoter region, suggesting a tightly coordinated expression (4). *MatPi* is on the same strand as *matMc*, although 8 kb away. Current findings suggest that the three MAT genes of Pneumocystis spp., *matMc*, *matPi*, and *matMi*, are sufficient to initiate mating and meiosis (4). In the present study, *matMc* gene expression was elevated after 2 weeks of treatment with anidulafungin, then decreased for the remaining 4 weeks of treatment (Fig. 3). In contrast, *matMi* remained downregulated during the 6 weeks of treatment and thus did not share the same expression profile as *matMc*, which is not in keeping with a predicted coordinated expression. The expression of *matPi* was the only MAT gene to be upregulated, if only at 4 to 5 weeks of infection.

Pneumocystis spp. lack a critical gene for a P mating type (*matPc*) but do have the genes associated with a M mating type that appears to display both mating receptors (encoded by *mam2* and *map3*) (4). Such a scenario might result in all non-BG expressing organisms, presumably spores/trophic forms, being able to mate with each other without a second mating type present. The downregulation of the *mam2* receptor gene could then result in a paucity of these receptor proteins on the surface of the mating type, perhaps resulting in the inability to undergo the initial steps in the mating process.

The *gsc-1* gene encodes the only known 1,3-β-D-glucan synthase in Pneumocystis spp. (9). In the prophylactic model, this gene was upregulated at 3 weeks of infection and thereafter (Fig. 4). However, this increase did not correlate with the levels of BG in the lung which were dramatically below those of untreated P. murina and did not increase throughout the experiment (Fig. 2). This apparent contradiction begs further thought about the mechanism of action of the echinocandins. While this may appear to be in contrast to another study where micafungin was shown to ablate the *gsc-1* signal (normalized to host *hprt*) after treatment (10), this report differed from the present study by a shorter infection timeline, echinocandin regimen, and mouse model. Eddens et al. appeared to begin treatment after 4 weeks of infection with 3 mg of micafungin. In the present study, we inoculated the mice with a million P. murina based on all forms of the organism, not just asci. We treated the mice with 1 mg/kg of anidulafungin, 3 times per week for 3 weeks. Our mouse model was one of chronic immunosuppression with dexamethasone in the drinking water. Thus, the models used were quite distinct and could account for the differences in *gsc-1* expression.

In yeast, the *gas 4/5* genes are responsible for β-(1, 3)-glucanosyltransferase activity which is critical for the incorporation and remodeling of β-(1, 3)-glucan into the cell wall and for creating attachment sites for the anchoring of mannoproteins and chitin (11). In yeast, there are five separate gas proteins with the *gas5* gene being expressed during vegetative growth, while *gas4* is repressed during vegetative growth and expressed during sporulation (11). In our previous study which focused on differential gene expression in anidulafungin-treated versus untreated P. murina after 3 weeks of treatment, the P. murina homolog shared close homology to both the *gas4* and *gas5* genes from S. pombe and was the most highly expressed gene in that study (8). The kinetics of this gene in the present study were notable. By week 2, the *gas4/5p* gene was upregulated and remained in that state during the 6 weeks of the prophylactic study. The upregulation of both *gsc-1* and *gas 4/5* were mostly concordant in their increased expression after 2 to 3 weeks, yet the measurement of BG in the lungs of treated mice was clearly repressed (Fig. 2) at those time points and was in contrast to the expression of the two genes related to BG synthesis. The lack of BG in the P. murina from anidulafungin-treated mice was also shown by fluorescence microscopy with a labeled antibody specific to BG (1). What may be surmised from these data are that although the organisms are attempting to synthesize BG, presumably for asci production, there is a clear inhibition of this process. At this time, a potential mechanism has not been explored.

Lastly, the endoglucanase, *eng1*, was upregulated only during the last week of treatment. These enzymes hydrolyze internal 1,3-β-D-glucan linkages in polysaccharides, typically requiring a region of contiguous unbranched and unsubstituted 1,3-β-D-glucosyl residues as the substrate. Many fungal cell wall hydrolases have glucanase activity, and some of these enzymes also exhibit transglycosylase activity, that can break and reform bonds within and between polymers, resulting in remodeling of the cell wall. The Pneumocystis spp. *eng1* shares significant homology with the endo-1,3-β-D-glucanase from S. pombe where this enzyme facilitates cell separation and is associated with enzymatic cleavage of BG (12).

A possible explanation for these results may be that because the process of BG synthesis was halted, the remaining organisms were attempting to remodel any remaining BG for the purpose of continuing through the mating process.

In S. pombe, there are three *FKS* homologues, with similar activities for *FSK1* (Bgs1p) and *FSK 2* (Bgs2p) and an essential role for germination and vegetative growth by *FKS3* (Bgs3p) (13). Although there is no genetic system available to knock out genes in Pneumocystis spp., extended treatment with the long-acting echinocandin, Rezafungin, resulted in eradication of Pneumocystis pneumonia in mice (7), also suggesting BG is essential for survival of Pneumocystis spp. as it is in yeast. Sporulation is effectively halted by treatment with echinocandins and asci are absent. The dysregulation of the mating type receptors may indicate there could be a feedback system that indicates a lack of BG availability or synthesis, resulting in a halt of conjugation. It has been suggested that Bgs3p may be involved in the transport of 1,3-β-D-glucan across the plasma membrane (13), and if the Pneumocystis Gsc-1p has combined activities of these yeast homologs, it could presumably act as a sensor itself.

### Suppression of asci by anidulafungin in treated mice prevents infection when transferred to treated recipient mice.

**FIG 6:**
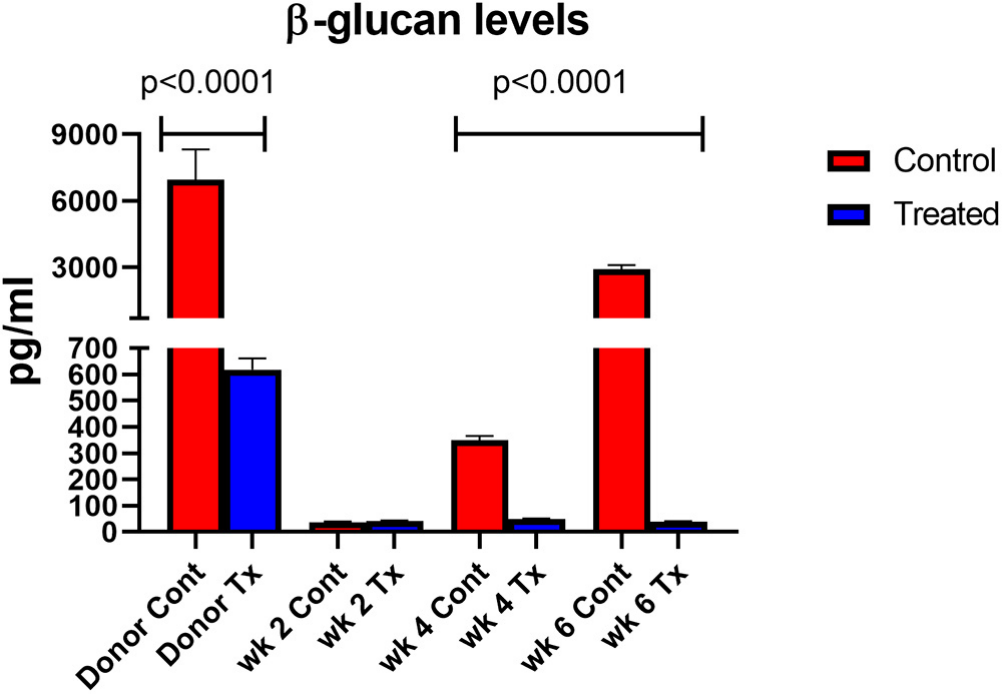
BG levels of mice that were inoculated with P. murina from anidulafungin-treated (1 mg/kg/wk) and untreated mice. Red bars = controls; blue bars = treated. Mice were sacrificed at 2-week intervals postinoculation. At each time point, three mouse lungs were homogenized for quantification of BG.

We have previously shown that anidulafungin-treated mice without asci cannot transmit the infection via an airborne route of infection. However, when organisms from these anidulafungin treated mice were inoculated into uninfected immunosuppressed mice, the treated organisms were able to cause infections that were replete with asci. Here, we asked whether P. murina from anidulafungin-treated mice could cause infection when inoculated into uninfected immunosuppressed mice that received anidulafungin treatment. The rationale was 2-fold: (i) to evaluate whether an alternative method of replication could maintain low levels of infection when exposed to anidulafungin and (ii) confirm that the production of BG and asci is necessary for survival of P. murina. Mice were inoculated with P. murina and immunosuppressed for 5 weeks. One group of mice then received 3 weeks of anidulafungin (“Donor Tx”) and another group received no drug (“Donor Cont”). Mice were sacrificed and the organisms from the Donor Tx (treated) mice were inoculated into immunosuppressed mice that were then administered anidulafungin to suppress the sexual cycle (Tx). Organisms from untreated mice (Donor Cont) were inoculated into untreated immunosuppressed mice as a control (Cont). Mice in both groups were then sacrificed at 2-, 4-, and 6-weeks postinoculation, and assessed for organism burden and BG content. Untreated control mice produced asci and nuclei by 4 weeks after inoculation whereas the P. murina from anidulafungin treated mice were unable to produce an infection (Fig. 5). The lack of asci in the treated mice never varied over the 6 weeks, while the number of non-BG expressing organisms from these same mice dropped to undetectable levels by the second week and remained as such over the 6-week period. Though BG was detected in the Donor Tx inocula (615 pg/mL) (Fig. 6), it was significantly less than in the untreated inocula Donor Cont (6950 pg/mL).

**FIG 7:**
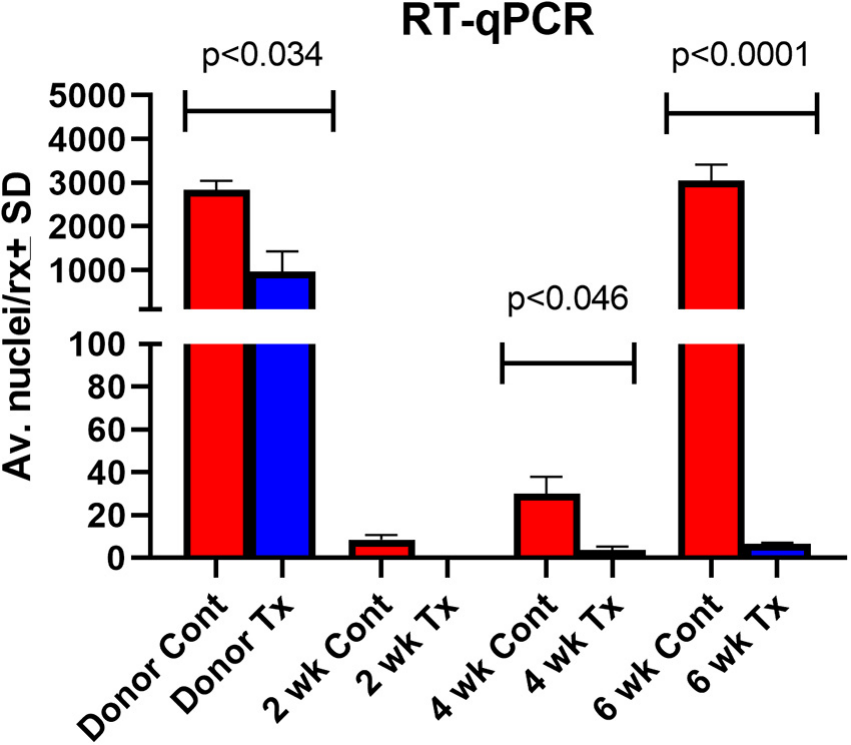
RT-qPCR of P. murina rRNA from treated and untreated mouse lungs after inoculation of anidulafungin treated and untreated donors. “Donor Cont” (red bar), from immunosuppressed mice that received the untreated inocula and “Donor Tx” are results from anidulafungin treated mice at day 0. Mice from each group were sacrificed at 2-, 4-, and 6-weeks postinoculation (Cont; Tx). Significance was calculated for differences between control and treated mouse groups. Capped lines indicate significant differences between control and treated groups.

Real-time reverse transcription-PCR (RT-qPCR) directed to P. murina rRNA gene expression from the Donor Tx inocula showed significantly reduced signal than that in untreated inocula (Donor Cont) (Fig. 7). As in BG levels that were undetectable, there was no detectable RT-qPCR signal 2 weeks after inoculation in the anidulafungin group. The repression of sexual reproduction vis-à-vis asci production resulted in a lack of growth and survival of the P. murina in the lungs of its mammalian host.

## DISCUSSION

Echinocandin treatment in most susceptible fungi leads to death due to inhibition of BG synthesis. Infections with Pneumocystis spp. are distinct from other fungal infections such that there are two major forms displayed; trophic forms which do not contain BG, and asci which do contain it. Echinocandins target the asci, initiating the breakdown of the cell wall followed by a disappearance of asci. However, even after 3 weeks of treatment, large organism burdens that do not express BG remain and do not appear to increase. Inoculation of these organisms into recipient mice produced a pneumonia replete with asci, indicating that the 3-week treatment was not sufficient to eradicate the infection (1).

However, we recently showed that prolonged treatment (8 weeks) with Rezafungin, a long-acting echinocandin, led to eradication of the infection in mice and also prevented the infection in the same prophylactic model used in the present study (7). While it was suspected there could be alternative methods of replication, e.g., asexual, the lack of infection in anidulafungin-treated recipient mice that received P. murina from anidulafungin-treated mice showed that there is not an alternative mode of replication these fungi can use when the sexual cycle is halted by treatment with an echinocandin.

We are learning that echinocandin treatment in Pneumocystis spp. has both downstream and upstream effects on the sexual cycle of these fungi. Downstream effects include the lack of asci and ascospore development and the inability to transmit the infection to a new host (1). New to our understanding are the upstream effects in the sexual cycle which appear to inhibit the process of mating, as indicated by the dysregulation of genes associated with this process.

Most fungi are facultative sexual species, meaning that they do not rely on this mode for survival, but rather on an asexual mode that can be less costly to the fungus. The findings herein set the genus Pneumocystis apart from most other fungi in that respect. Evolutionarily, the sexual cycle is thought to have arisen in response to environmental stresses to repair DNA damage and the maintenance of meiosis in fungi appears to be vital for success as it is also a source for genetic variation (14). Pneumocystis spp. inhabit a hostile environment in the mammalian lung, their primary environment, where they are exposed to the host immune system, and thus could employ the sexual cycle for both repair of DNA damage and genetic variation.

While other human pathogens in the Ascomycota and the basidiomycete, Cryptococcus neoformans, eschew sexual replication in their host, at least one host-obligate fungus, C. albicans, has evolved a unique mode, parasexuality, to circumvent the host immune responses (15). The immune responses are thought to be the primary reason that most pathogenic fungi do not undergo sexual replication in their hosts (although they have the genetic requisites) because products, such as ascospores, are highly antigenic and elicit a robust immune response (15). Pneumocystis spp. may also have evolved another way in which to evade most of the immune responses. The trophic forms outnumber the asci at an estimated 10:1 ratio, and sometimes by an even greater ratio (16). The trophic forms are covered in a family of variable surface glycoproteins (major surface glycoproteins [MSG]) and do not express the proinflammatory factor, 1,3-β-D-glucan, while asci are the only life cycle stage that do express this pro-inflammatory factor and are far fewer in number. The genes encoding the MSG proteins comprise 3% to 6% of the total genome, and these proteins are thought to help evade the host immune response by concomitant expression of closely related Msg proteins, which may lead to antagonism, reducing the normal activation (17). On the other hand, CLR Dectin-1 is the primary fungal β-glucan receptor in fungi and plays an important role in the clearance of Pneumocystis organisms (18). The MSG family of genes are located on the subtelomeric and telomeric ends of the linear chromosomes of Pneumocystis (19). Notably, a single MSG isoform is transcribed from a discrete expression site on a single chromosome where an upstream conserved sequence (UCS) resides (20). Gene conversion is thought to be responsible for the apparently limitless variation of these glycoproteins, whereby an MSG at a silent locus could donate some or all of its sequence to the UCS-linked MSG gene in a nonreciprocal manner (20). Such a process, integral to the survival of these fungi, would be facilitated by sexual reproduction (4).

While the studies presented here call into question the long-held notion that Pneumocystis spp. have an asexual cycle, it should be considered that such a cycle may be present in an infection, but to serve a different function (6). Transmission electron micrographs consistently show the trophic forms in tight apposition with the alveolar epithelial cells type 1 (AEC1). It has been suggested that these might be the vegetative cells, gathering nutrients from the host and its environment. Perhaps these cells require such a state prior to becoming mating competent, but do not replicate or if they do, it is not essential. As an alternative, it is tempting to speculate that their tight adherence to the AEC1 cells alters the environment, possibly by inducing a local hypoxia for these fungi that do not have genes to encode carbonic anhydrases (21). Carbonic anhydrases have been shown to be essential for virulence, growth, or acclimatization in other fungi and parasites (22).

The studies presented here further our understanding of the life cycle of these enigmatic fungi, but other questions remain. Asci via sexual replication appear to be vital to the growth and viability of Pneumocystis spp. and this process could be the key to a successful *in vitro* culture system, which has thus far evaded efforts to establish.

## MATERIALS AND METHODS

### Animals.

Male BALB/c (Charles River, Raleigh, NC) mice were handled in strict accordance with good animal practice, as defined by the University of Cincinnati and Veterans Affairs Medical Center Institutional Animal Care and Use Committee (IACUC). The animal husbandry and experimental procedures were consistent with the recommendations in the Guide for the Care and Use of Laboratory Animals, the Animal Welfare Act Regulations, and the Public Health Service Policy on Humane Care and Use of Laboratory Animals. To safeguard against environmental exposure of P. murina and other microbes, mice were housed under barrier conditions with autoclaved food, acidified water, and bedding in sterilized shoebox cages equipped with sterile microfilter lids. Access was limited to animal care and technical personnel, who were required to wear sterile caps, gowns, masks, gloves, and shoe covers while in the animal rooms.

### Study 1 experimental design, prophylactic model.

Mice (5 to 6 weeks of age) were intranasally inoculated with 10^6^ P. murina nuclei which contained all life cycle stages, immunosuppressed by the addition of dexamethasone (4 mg/L) in acidified drinking water and intraperitoneally injected with 1 mg/kg of anidulafungin (Eraxis-Pfizer, New York, NY) once per week. The mice in the control group were inoculated and immunosuppressed as detailed above but received no anidulafungin treatment. Mice were then sacrificed weekly for 6 weeks. At sacrifice, three mouse lungs were homogenized for quantification of P. murina asci, nuclei and 1,3-β-D-glucan (BG) content. Three additional mouse lungs were snap-frozen in liquid nitrogen for gene expression analysis by RT-qPCR.

### Study 2 experimental design, treatment model.

Mice were inoculated and immunosuppressed as stated above. After a period of 5 weeks when the infection reached moderate organism burdens, mice were divided into control and treatment groups and the treated group was begun on the anidulafungin regimen of 1 mg/kg of anidulafungin 3 times per week for 3 weeks. At the end of treatment, the right lung from three control and three treated mice were homogenized for quantification of P. murina asci, nuclei, and BG content. The left lungs of these mice were snap-frozen in liquid nitrogen for quantification of P. murina by qRT-PCR. The remaining control and treated mouse lungs were homogenized and P. murina from these mice was then inoculated into naive immunosuppressed mice. The mice receiving the treated P. murina inocula were continued on the anidulafungin-treatment regimen, while the control mice remained untreated but immunosuppressed. Control and treated mice were then sacrificed at weeks 2, 4, and 6, and processed same as above.

### Microscopic quantification of organism burdens.

Lungs were dissociated in 10 mL of phosphate-buffered saline (PBS) by means of a gentleMACS Tissue Dissociator (Miltenyi Biotec, Auburn, CA). The lung tissue was then filtered through a 40-μm-pore mesh, and P. murina was recovered by centrifugation at 3,400 × *g* for 10 min and reconstituted in 2.0 mL of PBS. Slides were prepared with 3 × 10 μL of the homogenate placed onto pre-etched glass microscope slides (Thermo Fisher Scientific Co., Cincinnati, OH), allowed to air dry, and the slides were then heat fixed and stained with either Cresyl Echt Violet (CEV) to enumerate asci or Hema 3 (Thermo Fisher Scientific Co.), a rapid variant of the Wright-Giemsa stain, to enumerate nuclei for the non-BG expressing life cycle stages. P. murina asci and nuclei were enumerated by counting the numbers of each form in 30 microscopic fields under oil immersion (1,250× power). The microscopic counts were log transformed, and values were compared by the one-way analysis of variance (ANOVA) followed by Newman-Keuls multiple-comparison posttest using GraphPad Prism v8 (GraphPad, San Diego, CA). Significance was accepted when the *P* value was <0.05. The limit of detection by this method is 1.75 × 104 (log_10_ 4.24/lung).

### Measurement of 1,3-β-D-glucan (BG) content.

The same homogenate used for microscopic enumeration of P. murina was used to measure BG content. The homogenates are reconstituted in 2-mL PBS and aliquots of 10 μL are diluted to 1:10 and 1:100. A Glucatell assay kit (Associates of Cape Cod East Falmouth, MA) was used according to the manufacturer’s instructions. Homogenates were pretreated in 0.125 M KOH and 0.6 M KCl for 10 min to eliminate endogenous proteases and unwind the triple helical 1,3-β-D-glucan structure to single helices. Samples were prepared in 10-fold dilutions in pyrogen-free water. BG content was measured on a Synergy HTX microplate reader (BioTek, Winooski, VT) by using a linear regression curve of standards and statistical analysis was performed comparing values by *unpaired t test* using GraphPad Prismv8. Data are expressed as pg/mL.

Note: GlucaTell is used for research purposes. The Glucatell kit is specific for detection of 1,3-β-D-glucan (BG) and is used for detection in food sources and other nonserum samples. FungiTell is FDA-cleared and CE marked rapid *in vitro* diagnostic screening test for invasive fungal infections in serum.

### RT-qPCR quantification of mating-associated gene expression levels.

RNA was isolated from lungs that were snap-frozen in liquid nitrogen using TRIzol reagent (Life Technologies, Grand Island, NY) per the manufacturer’s protocol. The RNA was treated with RQ1 DNase (Thermo Fisher Scientific) and recovered by a phenol: chloroform extraction and ethanol precipitation. cDNA was synthesized from 1 μg of the extracted RNA by Superscript II reverse transcriptase (Invitrogen, Carlsbad, CA) per the manufacturer’s protocol. The remaining RNA and synthesized cDNA were stored at −80°C.

Primers were designed to span an intron juncture to prevent amplification of genomic DNA (see Table 1). RT-qPCR was performed in an Applied Biosystems 7500 fast real-time PCR system (Life Technologies, Grand Island, NY). The reactions were performed in triplicate in a final volume of 20 μL containing 1× PowerUp SYBR green master mix (Life Technologies, Grand Island, NY) and 500 nM each primer pair. The reaction mixtures were initially incubated at 50°C for 2 min and 95°C for 2 min, followed by 40 cycles of 15 s at 95°C, 59°C for 15 s, and 72°C for 1 min. Fluorescence data were captured during the 72°C step. Disassociation curves were also calculated for the reactions to determine the melting temperature (*Tm*) of the products. The threshold cycle (Δ*CT*) value between the validation gene and the thymidylate synthase (TS) reference gene was calculated by subtracting the average *CT* value of the single-copy reference gene from the average *CT* value of the validation gene. ΔΔ*CT* values were calculated by subtracting the Δ*CT* value of the reference sample from the Δ*CT* value of the experimental sample. Relative quantity is shown as 2^_ΔΔ^*^CT^*.

**TABLE 1:** Primers used for gene expression analysis

Target	Direction	Primer sequence 5′→3′
*gsc1*	Forward	TGGTTTTGGTGGTGGTGTTG
	Reverse	CGACGCTGACATTCGGTTTC
*gas4/5p*	Forward	TCATGGTGTTCTCCTTCCTCAT
	Reverse	GGCCTTGGGACTGCTCTATT
*eng1*	Forward	CACCTAGATTTTCAGGGATTCCTTCATTAG
	Reverse	AAGAGGTTCTCGAGTAGTAATAGGAGTAAG
*mam2*	Forward	TGGCACTTGTCCTTATGTTGAC
	Reverse	AGGTGCCTGACAAATAAGCA
*map3*	Forward	CGATCATGGCTAGCTGTG
	Reverse	CATTTCGCTTTTTGAAGTATG
*matMc*	Forward	GAATCCTCCACGACCACCTA
	Reverse	TCGCTGTTTTACAGCTGGTG
*matPi*	Forward	CAACAAGGAATTGTCGGAGAC
	Reverse	TCCGACATAAATCCGACAGA
*matMi*	Forward	GAAATGGAGACATTGGAGAGGAAG
	Reverse	TATCCGACTCACGAAATATAGCATC
*TS* ^*a*^	Forward	CTTTCAGCATGGAATCCTTCAG
	Reverse	CTAGCCCCATGTCACAAGAAC
*LSU rRNA* ^*b*^	Forward	ATGAGGTGAAAAGTCGAAAGGG
	Reverse	TGATTGTCTCAGATGAAAAACCTCTT
	Internal	/56-FAM/-AACAGCCCAGAATAATGAATAAAGTTCCTCAATTGTTAC-/36-TAMSp/

a Thymidylate synthase.

b Large subunit rRNA.

### RT-qPCR quantification of P. murina rRNA expression levels.

cDNA from frozen mouse lung was synthesized same as above. Quantitation of the amount P. murina rRNA gene message in the samples was performed on the 7500 Fast real-time PCR system (Applied Biosystems, Foster City, CA) using a previously described TaqMan assay (23). The threshold cycle for each sample was identified as the point at which the fluorescence generated by degradation of the TaqMan probe increased significantly above the baseline. To convert the threshold cycle data to P. murina nuclei, a standard curve was generated using cDNA made from RNA isolated from 10^7^ P. murina nuclei. The level of infection of the samples was then estimated using the standard curve. The efficiency of the standard curve qPCRs consistently approached 100%.

To ensure that high-quality RNA was isolated from all samples and that cDNA synthesis was successful, a SybrGreen incorporation qPCR assay for the mouse GAPDH gene mRNA was performed on all samples. The fluorescent signal generated by incorporation of SybrGreen into the double-stranded product was collected to determine the threshold cycle for each sample. The fidelity of the qPCR reactions was confirmed by analysis of the melt curve of the GAPDH qPCR product.
